# The socio-environmental determinants of railway suicide: a systematic review

**DOI:** 10.1186/1471-2458-14-20

**Published:** 2014-01-10

**Authors:** Lay San Too, Allison Milner, Lyndal Bugeja, Roderick McClure

**Affiliations:** 1 Monash Injury Research Institute, Monash University, Melbourne, Victoria 3800, Australia; 2 McCaughey VicHealth Centre for Community Wellbeing, Melbourne School of Population and Global Health, The University of Melbourne, Melbourne, Victoria 3010, Australia

## Abstract

**Background:**

Railway suicide has significant adverse impacts for the victims, their family and friends, witnesses to the incident, general public and train network. There is no previous review on the socio-environmental factors and railway suicide. The research question asked in this review was: *‘What socio-environmental risk and protective predictors are significantly associated with railway suicide?’*

**Methods:**

The review searched Medline, PsycINFO, Web of Science and Scopus for English-language studies that assessed the associations between socio-environmental (i.e. geographical, physical, economic and social) factors and railway suicide from their inception to June 2013. It was reported based on the PRISMA Statement.

**Results:**

Eleven studies met the inclusion criteria. They were categorised into railway environments (availability of railways and trains, accessibility to railways and familiarity with trains), population characteristics and impact of media reporting. Findings from ecological studies using population level railway suicide data suggested weak and inconsistent evidence for the first two categories. The evidence on the impact of media reporting was moderately strong, with irresponsible media reporting being associated with an increased risk of railway suicide.

**Conclusions:**

There is a need for further research activity to strengthen evidence about socio-environmental risk factors for railway suicide. The focus of this research should be on the factors that determine individuals’ decisions of using the railway as a method of suicide, with the consideration of a range of geographical, physical, social, and economic factors.

## Background

Railway suicide accounts for 1 to 12% of all suicides globally, with up to 94% of all attempts being fatal [1]. International studies show that over half of all rail-related fatalities are suicides [2-4]. The direct adverse impacts of railway suicide for the victim can include death and significant physical disability. The trauma of railway suicide also affects family, friends, and relatives of the deceased, as well as witnesses to the suicide incident, particularly the train driver, railway staff and passengers [5,6]. Other major consequences for the general public and train network include financial losses through delays and cancellations of rail services and driver absenteeism [6,7]. Railway suicide is also reported to trigger imitative behaviour (i.e., at-risk individuals may use the same suicide method after exposure to an incident) because trains are a known public infrastructure [8].

Suggested prevention strategies for railway suicide have emphasised the need to decrease the perceived attractiveness (e.g. increase public knowledge on survival rate) and availability of trains as a suicide means (e.g. reduce train frequency) [9]. These strategies have also suggested reducing accessibility to train lines (e.g. install physical barriers) and the potential of collision (e.g. decrease train speed), mitigating the consequences of collision (e.g. modify the front design of the train), and increasing medical survival and recovery (e.g. offer rehabilitation for survivors) [9]. As it stands, reducing accessibility to train lines through installation of physical barriers is the only suicide prevention strategy that has been followed by a decrease in railway suicide [10]. These findings align with several reviews on limiting access to ‘lethal’ methods via physical restriction and a reduced number of suicides, with minimal substitution effect to other suicide method [10-16]. The fundamental assumption underlying method restriction (i.e. restricting access to railway) is that, at an individual level, suicide crises are often short-lived. Many at-risk individuals have a preference for a particular method, which would limit the likelihood of substitution to another method [17,18]. There is some support for this assumption. For example, a ten year follow-up study of 94 railway suicide attempters found that only 7% of these persons went on died by suicide and 3% repeated the same method three and half years after their initial attempts [19].

Nevertheless, the application of method restriction may not always be feasible and cost-effective, particularly if railway networks cover a large area [20,21]. Further, this strategy ignores the role of other factors that may increase the risk of railway suicide in a geographical area, such as socio-economic disadvantage, which has been shown to be related to elevated rates of general suicide [22,23]. While such knowledge is useful to characterise high-risk locations for railway suicide; currently, no systematic review has specifically examined socio-environmental factors and railway suicide. This systematic review seeks to address this gap by examining the existing evidence on the socio-environmental predictors of railway suicide. Specifically, it addresses the following research question: ‘*What socio-environmental risk and protective predictors are significantly associated with railway suicide?*’

## Methods

**Figure 1:**
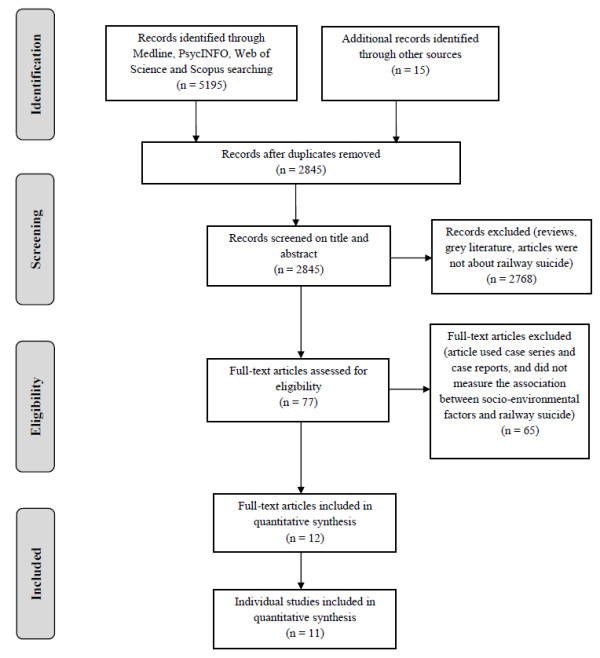
PRISMA flow diagram for the selection of studies.

This review was conducted in accordance with the Preferred Reporting Items for Systematic Reviews and Meta-Analyses (PRISMA) Statement [24] (see Figure 1 and Additional file 1).

### Search strategy

The search was performed using four databases: Medline (Ovid), PsycINFO (ProQuest), Web of Science (Web of Knowledge) and Scopus (SciVerse) from their inception to June 2013. These databases were selected because they are the main databases for research in the fields of behavioural sciences, social sciences and medicine. The following search terms mapped onto title, abstract or keywords were used: (suicid* OR self injur* OR self harm) AND (rail* OR train OR subway OR underground OR metro OR tube OR violent OR moving object). These terms were obtained from other review papers about railway suicide [1,25,26] and the relevant code of the 10th revision of the International Statistical Classification of Diseases and Related Health Problems (ICD-10). We sought only English language journal articles.

To identify additional relevant articles, references of main review articles and included studies were reviewed. One author (LST) conducted the initial searches (identification and screening). Two authors (LST and AM) assessed the full-text articles for eligibility and inclusion, with inconsistency in classification resolved by consensus.

### Inclusion and exclusion criteria

This review only included original research articles published in journals. Editorials, commentaries, conference proceedings, working papers, reports and review articles were excluded. We included studies with an outcome variable of fatal (suicide) and non-fatal (suicide attempt) railway suicide, and exposure variables of socio-environmental factors. In terms of socio-environmental factors, we specifically examined geographical, physical, economic and social characteristics for railway suicide such as: railway- and train-related factors (e.g. track length, train frequency, number of train passenger, physical barrier, surveillance measure, and blue light), population characteristics, and presence of media reporting on railway suicide. We considered only studies that measured associations such as ecological, cross-sectional, cohort, case control, quasi-experiment, non-randomised control trial and randomised control trial. Case series studies and case reports were excluded.

### Data extraction

Two authors (LST and RM) reviewed the full text of the papers that met the inclusion criteria and extracted the following information:

• author(s) and date of publication

• setting

• study design

• population/sample and observation period

• outcome variables

• socio-environmental factor(s) of interest

• significant relationships between study factors and outcomes

Inconsistency in the information extracted by the authors was resolved by consensus. Contact with one author was made to clarify the definition of one variable.

## Results

The process of how the studies selected for inclusion in quantitative synthesis is demonstrated in Figure 1. The primary search yielded 77 potential articles. Of these, 12 articles describing 11 studies met the inclusion criteria [27-38] (Table 1). Among the 65 articles that were excluded, four articles described railway suicide preventive measures, one article measured the reliability of railway suicide rates, 27 articles described only the individual and temporal factors related to railway suicide, and 33 articles described the socio-environments that were relevant to railway suicide using case report and case series methods.

**Table 1:** Study characteristics and findings

**Study Number**	**Author(s) (Date)**	**Setting**	**Study design**	**Population/sample and observation period**	**Outcome variables**	**Socio-environmental factor(s) of interest**	**Significant relationships between study factors and outcomes**
**Non-intervention studies**							
1	van Houwelingen, C., et al. (2013) [27]	Railway systems in the Netherlands and Germany	Ecological study, comparing Dutch and German railway suicides in terms of railway and population parameters.	1475 Dutch and 6105 German railway suicides in 2000 – 2007	Railway suicides	• Railway length	Positive association:
						• Train frequency	• Train frequency (RR = 0.74)
						• Number of train passengers	• Population density (RR = 0.59)
							No association:
						• Population density	• Railway length (RR = 1.49)
							• Number of passengers (RR = 1.20)
2	Hegerl, U., et al. (2013) [28] & Ladwig, K.–H., et al. (2012) [29]	Railway system, Germany	Quasi experimental, ecological study, comparing railway suicidal behaviours before and after the railway suicide incident.	310 railway suicidal behaviours in index period of 2006–2008, 188 in index period of 2009 (Index period = 11th November to 31st December of the year).	Railway suicides and suicide attempts	Railway suicide of the famous German football goal-keeper.	• Railway suicidal behaviours in the index period increased by 1.81 (IR), after adjusted for daily temperature.
							• Railway suicidal behaviours increased by 2.2 (IR) after 28 days of the incidence, after adjusted for daily temperature.
							• Railway suicidal behaviours increased by 18.8% in the following two years.
3	Niederkrotenthaler, T., et al. (2012) [30]	Subway system, Vienna, Austria	Ecological study, measuring the associations between station characteristics and subway suicidal behaviours.	107 subway suicide attempts and 185 subway suicides in 1979 – 2009	Railway suicides and suicide attempts	• Presence of a surveillance unit	Positive association:
						• Train types	• Presence of a surveillance unit (RRs = 1.65, RRa = 1.93)
						• Station construction on the street level versus other construction	
						• Distance between stations	• Faster train (associated with railway suicides after full adjustment RRsf = 3.53, but not associated with railway suicide attempts)
						• Distance between a station to historical sites	• Number of stations operated in extensive media reporting period (RRs =1.82, RRa = 1.71)
						• Number of stations operated in the years of extensive media reporting	• Drug scene (RRs = 1.49, RRa = 2.56)
						• Number of train passengers	• Number of train passengers (RRs = 1.02, RRa = 1.03)
							• Distance between a station to historical sites (only for railway suicide attempts, RRa = 1.78)
						• Station used as local drug scene	No association:
						• Socioeconomic status of the population in the station’s neighbourhood	• Station construction
							• Distance between stations
							• Socio-economic status of station’s neighbourhood
4	Kunrath, S., Baumert, J., & Ladwig, K-H. (2011) [31]	Railway system, Germany	Quasi experimental, ecological study, comparing railway suicidal behaviours before and after the presumed railway suicide incident.	747 railway suicidal acts from December to March in 2004–2007 Index period: first 2 months after the presumed railway suicide incident (January and February 2007).	Railway suicides and suicide attempts	Media coverage of a presumed railway suicide on the main railway trunk line following by 3 investigators killed by an express train.	• Daily railway suicidal behaviours increased by 44% following extensive media coverage of the presumed railway suicide incident in the index period, after controlling for unemployment rates and temperature.
5	Van Houwelingen, C. A. J., Kerkhof, A. J. F. M., & Beersma, D. G. M. (2010) [32]	Railway system, the Netherlands	Ecological study, measuring the associations between railway and population parameters and railway suicide rates.	5178 railway suicides and 517 railway suicide attempts in 1980–2007	Railway suicides and suicide attempts	• Railway length	Positive association:
						• Train mileage	• General suicides (similar trend)
						• Passenger kilometre	No association:
							• Railway length
						• Free transport for students	• Train mileage
						• Population density	• Passenger kilometre
						• General suicides	• Free transport for students
						• Presence of high risk populations near railways	• Population density
6	Baumert, J., Erazo, N., & Ladwig, K. H. (2006) [33]	Railway system, Germany	Ecological study, measuring the associations between availability of railway and train and railway suicide trend.	8653 railway suicides and 857 railway suicide attempts in 1991-2000	Railway suicides and suicide attempts	• Railway length	For subjects aged ≤ 65 years old,
						• Train mileage	Positive association:
						• Passenger kilometre	• Railway length (AAPC = 3.2)
							Negative association:
							• Passenger kilometre (AAPC = −2.7)
							No association:
							• Train mileage
							For subjects > 65 years old,
							Negative association:
							• Train mileage (AAPC = −8.8)
							• Passenger kilometre (AAPC = −10.4)
							No association:
							• Railway length
7	Clarke, M. (1994) [34]	Railway system, England and Wales, the United Kingdom	Ecological study, measuring the associations between availability of railway and train and railway suicide	4171 railway suicides in 1852-1947	Railway suicides	• Growth of railway length	Positive association:
						• Number of train passengers	• Growth of railway length
							• Number of train passengers
8	Schmidtke, A., & Häfner, H. (1988) [35]	Railway system, Germany	Quasi-experimental, ABABA design (A = baseline phase, B = broadcasting phase)	Years 1976–1984. First broadcasting in 1981, second broadcasting in 1982.	Railway suicides	Twice-broadcast six-episode weekly serial showing the railway suicide of a 19-year-old male student.	• Railway suicides increased by 175% in the period during and just after the two broadcasts, for the group whose age and sex were most similar to those of the film model.
**Intervention studies**							
9	Matsubayashi, T., Sawada, Y., & Ueda, M. (2012) [36]	71 metro train stations, Japan	Quasi experimental, ecological study, comparing railway suicide number pre- and post-installation of blue light.	The average number of suicides per station-year observations was 0.164 in 2000-2010	Railway suicides	Installation of blue light in 11 stations with blue light and 60 stations without blue light	• Railway suicides decreased by 84% after installation of blue lights, after controlling for the number of suicides in the previous years, use of faster train, proximity to a psychiatry hospital.
10	Law, C. K., et al. (2009) [37]	Underground system, Hong Kong	Quasi-experimental, ecological study measuring railway suicide before and after the installation of platform screen door.	76 railway suicides in 1997-2007	Underground railway suicides	Installation of platform screen door in 30 stations in year 2002	• Railway suicides decreased by 59% since the installation of platform screen door, adjusted for age and gender.
							• No sign of suicide substitution to unsealed platforms.
							• Railway suicides of deceased with psychiatric profile decreased by 84%.
11	Niederkrotenthaler, T., & Sonneck, G. (2007) [38]	Subway system, Vienna, Austria	Quasi experimental, ecological study, measuring subway suicide before and after the introduction of media guidelines.	Year 1982/83 to 2004/5	Subway suicides	Introduction of media guidelines in 1987/88.	• Subway suicides decreased by approximately 62 cases following the introduction of media guidelines, after controlling the passenger number.

*RR* Dutch-German rate ratio; *IR* incidence ratio; *RRs* crude rate ratio for railway suicide; *RRa* crude rate ratio for railway suicide attempt; *RRsf* rate ratio for railway suicide after full adjustment; *AAPC* average annual percentage change of the number of suicide.

### Study characteristics

The included eleven studies were based in six countries: Germany, Austria, Netherlands, the United Kingdom, Japan and Hong Kong. Four studies examined metro, subway or underground suicide and seven studies considered suicides occurring in railway systems (including two studies that did not provide information on whether the suicide data of railway systems included or excluded metro suicides [34,35]). Most of the studies were conducted in recent years (2006–2013) while only two studies were published before year 2000. Ten studies used an ecological approach to examine the relationship between socio-environments and railway suicide. Of these, five studies combined ecological approach with quasi-experimental method. The remaining one study applied only quasi-experimental method. Eight studies were non-intervention studies while the other three studies examined the impact of an intervention on railway suicide. Half of the selected studies measured outcomes of both fatal and non-fatal railway suicidal outcomes while the other half measured only fatal outcomes. The socio-environments studied were categorised as railway environments, population characteristics and impact of media reporting.

### Railway environments

Seven studies investigated whether railway- and train-related parameters were correlated with railway suicide [27,30,32-34,36,37]. These parameters included the availability of railways and trains (e.g. track length, train frequency, train mileage, fast train), accessibility to railways (e.g. presence of a surveillance unit, platform screen door and blue light, street-level station construction, distance between stations, distance between station and historical sites), and familiarity with trains (e.g. number of passenger, distance travelled by passengers, free transport for students).

The observed association between availability of railways and trains with railway suicide differed between countries. A study conducted in England and Wales reported a positive correlation between the number of railway suicide and track length [34]. A similar finding was also found in suicide victims aged ≤ 65 years old but not in those aged > 65 years old in Germany [33]. However, another study comparing Dutch and German railway suicides observed that track length had no effect on the incidence of railway suicide but that train frequency did [27]. The study suggested that the higher train frequency explained the higher railway suicide rate in the Netherlands. No association between track length and the frequency of railway suicide was confirmed by a study based in the Netherlands [32]. This study also showed no correlation between train mileage and railway suicide rates [32]; whereas, another study reported a negative relationship between train mileage and railway suicide victims aged older than 65 years old in Germany [33]. An ecological study that examined train types and subway suicidal acts in Vienna found that suicide deaths but not suicide attempts significantly increased when stations were served by the faster train type, even after full adjustment of confounders [30]. These findings concluded that availability of trains (e.g. higher train frequency and fast train) is more predictive of railway suicide compared to availability of railways (e.g. longer track length).

There was some evidence that limiting access to railways can prevent railway suicide. One study examined the effects of platform screen doors (i.e. total barriers between the station floor and ceiling that screen the platform from the train) on railway suicide in the underground system in Hong Kong over an eleven year period [37]. The study found that railway suicides significantly decreased (59.9%) after the installation of platform screen doors in 2002, suggesting that substitution to unsealed platforms had not occurred. In particular, the platform screen doors had a strong protective effect on victims who had history of mental disorder(s). Since 2006, blue lights (i.e. blue light-emitting–diode lamps stay on from sunset to sunrise) have been introduced to level crossings and later to train stations in Tokyo metropolitan area with the belief that it creates a calming effect. A team of researchers who have evaluated the effectiveness of blue lights on the risk of railway suicide found that an 84% reduction of railway suicides that could be attributable to the introduction of blue lights at the edges of stations [36]. Whether or not there was a substitution effect to the other stations without blue lights and to other means, was not explored. Contrary to expectations, the presence of surveillance units in stations was found to have no positive effect on railway suicide, with a higher frequency of railway suicides being observed in the subway stations with a surveillance unit compared to those without [30]. The construction of subway stations at the street level, compared to those not at street level, was not correlated with either fatal or non-fatal railway suicidal behaviours in Vienna [30]. The same study examined the distance between subway stations and the distance between subway stations and historical sites [30]. The results showed that the frequency of railway suicidal behaviours was not related to either of these proximity variables.

The number of train passengers and the distance travelled by passengers in kilometres has been used as an indicator for familiarity with trains. Two studies have demonstrated that the number of passengers and railway suicidal acts were positively correlated [30,34]. However, inconsistent findings were observed on the association between passenger kilometres and railway suicide rate. One study showed that more passenger kilometres was correlated with lower railway suicide rate [33]; whereas, two other studies reported no relationship [27,32]. Being familiar with railway transportation was also measured by introducing free transport for students [32]. It was found that this parameter was not correlated with the number of railway suicide among students aged 18–25 years old.

### Population characteristics

Three studies examined the relationship between the characteristics of the population and railway suicide [27,30,32]. One study found that population density (which reflects the number of people with potential physical exposure to railways) had a strong impact on rates of railway suicide [27]. Another study, conducted in the same country, did not find this impact [32]. As reported by one study, socioeconomic status in the subway station’s neighbourhood had no correlation with railway suicidal behaviours [30]. Some authors speculated about whether the presence of high risk populations near railways was associated with the higher number of railway suicide [30,32]. They reported that increased number of railway suicidal behaviours was found at stations used as meeting points by drug users [30]. Another study indicated that half of railway suicides occurred in a limited number of locations, which were close to a psychiatric hospital [32]. In relation to the rate of general suicide, railway suicide was found to shift in a similar pattern, particularly more prominent in females but not males [32].

### Impact of media reporting

Two papers explored the impact on railway suicide following the suicide of a famous German football goal-keeper, one on the short-term effect of the incidence while another on its long-term effect [28,29]. The study found that in Germany, suicidal behaviours on the railway increased significantly after 28 days, approximately two months, and two years following the railway suicide incident. The long-term effect was more pronounced in men than in women. This effect was not related to the unemployment rates in Germany. Consistent with this, another study showed that subway suicide increased when greater number of stations operated in the period of extensive media reporting of subway suicide [30].

In December 2006, a presumed railway suicide occurred on the train line between Munich in Germany and Zurich in Switzerland. Subsequently, three police were killed by an express train during the investigation. This traumatic incident received widespread attention in various media channels on that day and the following days. Following these events, researchers reported a 44% increase of daily railway suicidal acts within two months after the incident compared to the control periods, after adjusted for unemployment rates and temperature [31]. Another study set in Austria assessed the number of railway suicides over time since the introduction of responsible media reporting in mid-1987 [38]. It showed a significant reduction in railway suicides, after adjustment of the number of passengers, following the implementation of responsible media reporting. The changes of media reporting in the quality and quantity components also contributed to the overall decrease of general suicide.

In addition to the observed adverse consequences that resulted from the irresponsible media reporting of railway deaths (e.g. details on the suicide method are given), one study investigated the impact of broadcasting television program that showed the railway suicide of a 19-year old male student in 1981–1982 [35]. In comparison with the control periods, railway suicides in the period during and just after the two broadcasts rose significantly for the group whose age and sex were most similar to the film model. There was a considerable increase of the number of railway suicides among males aged 15 – 19 years old but no effect was found for males above 40 years old and females above 30 years old.

## Discussion

This review consolidates current knowledge about socio-environmental factors for railway suicide. There are relatively few studies addressing this topic, compared to the proportion of studies examining the epidemiology and individual characteristics of railway suicide. Virtually all studies included in the analysis used ecological design. Several of these studies were combined ecological approach with quasi-experimental method to evaluate the effects of particular measures. A causal relationship between exposure and outcome variables cannot be inferred from results of studies using these designs. Ecological methods are also subject to ecological fallacy (i.e. an error of inference that involves deriving conclusions about individuals exclusively based on aggregate level data) [39].

### Summary and interpretation of evidence

The existing evidence demonstrates that some socio-environmental factors are predictive for greater risk of railway suicide although they are determined by only one or two studies. For example, higher train frequency, increased number of train passengers, availability of fast train, and presence of high risk persons like drug users at station were found to increase risk of railway suicide [27,30,34]. In particular, the factors related to train frequency and number of train passengers were associated with suicides on the railway network [27,34] while the factors of availability of fast train, number of passengers and presence of drug users were associated with suicides in subway systems [30]. The strongest evidence relates to studies that have examined the association between irresponsible media reporting and railway suicide [28-31]. They consistently suggest that irresponsible media reporting was followed by a higher frequency of railway suicides. Only one study evaluated the impact of appropriate media guidelines and reported a positive effect [38]. There is also evidence on the correlation between railway suicide and general suicide in females but not in males.

The evidence is inconsistent on the relationship between suicides in railway systems and variables of track length, train mileage, distances travelled by train passengers and population density [27,32-34]. This might be explained by the diverse structure of railway networks across different countries and the use of various definitions/metrics of the same variables. For instance, track length was measured as the number of rail miles open in England and Wales [34], but it was measured as railway length in metres per kilometre square surface area in a study comparing Dutch and German railway suicides [27]. Another example was the distance travelled by train passengers. It was defined as passenger kilometres divided by the national population in one study [27] and the passenger volume per kilometre in another study [33].

Other factors such as station constructed at street level, distance between stations, proximity to station from historical sites, and free transport to student were not significant risk predictors for railway suicide. However, this evidence is very limited. Furthermore, the socioeconomic status of a population living in the neighbourhood of the subway station had no effect on railway suicide. A consistent finding was reported elsewhere, which showed that victims of railway suicide and other suicide did not differ in the economy deprivation status of their communities [40].

In addition to appropriate media reporting of suicide, factors observed to protect an individual from railway suicide include the existence of physical barriers such as platform screen doors [37] and the introduction of blue lights [36]. The impact of physical barriers on railway suicide was strong, without a substitution effect to unsealed platforms [37]. This is consistent with the conclusion derived from a recent systematic review [10], that the installation of barriers had the strongest evidence of effectiveness to decrease suicides at suicide hotspots. However, this measure is only applicable to underground railways or subways, but not to open railway networks. Furthermore, the introduction of blue lights is a new suicide preventive intervention that has only been examined in one location [36]. The longer-term effect of this measure, the underlying causal mechanism of why this is effective and substitution effect to other stations and other suicide methods over time should be evaluated.

### Future research

Given that the current knowledge is considerably inadequate, further research is warranted. While there is strong evidence that restricting access to lethal suicide method effectively prevents suicide [10-16], some researchers indicated that it is essential to understand the factors that influence selection of particular suicide method before any potential method restriction strategy is introduced [16]. They also suggested that not only physical availability of method play a crucial role in suicide method choice, cognitive availability (i.e. how accessible the method in one’s mind) can also be the key determinant [16]. There is still much research needed to enhance the knowledge of the factors influencing selection of railway as a suicide method, including assessment of a range of geographical, physical, social and economic factors (e.g. number of alcohol outlets and level of alcohol consumption in a geographical area because alcohol consumption may play a role in railway suicide [41,42]). There is also the need to introduce uniform measures of variables related to the railway network infrastructure and train traffic that are considered as important such as standardised measures for quantity of track length.

Ecological designs are commonly used to investigate socio-environmental determinants, but are limited by the ecological fallacy (as described above). Although the ecological level findings provide good starting points for further study, it could be strengthen using stronger observational designs (e.g. case control or cohort approaches) or triangulation of data from multiple sources if possible. For example, a multilevel model that measuring population-level data while controlling individual-level sources of bias can be implemented where practically possible.

### Limitations of the review

Although this review adopted a comprehensive search strategy, it has several limitations. We did not consider grey literature such as reports and conference abstracts. There may have been relevant studies conducted, but not published in a scientific journal. This is likely to be the case in the studies performed or funded by railway organisations where findings dissemination in a scientific journal is not recognised as important. Therefore, this review may be subject to publication bias.

This review did not include studies that examined temporal variations (i.e. seasons, days of week, time of day, and day or night time) because they were defined out of scope. We also excluded non-English articles. Because of this, it is possible that the review under-reports relevant studies conducted in non-English speaking areas of the world.

Another limitation of the review is that it included small number of studies, which were based in different countries with diverse structure of railway networks and applied various definitions in measuring same variables. All of these lead to making definitive conclusions difficult.

## Conclusions

Empirical evidence of socio-environmental factors for railway suicide is limited and inconsistent. It is not sufficient to determine appropriate strategies for the prevention of railway suicide. The review emphasizes that the focus of future research should be on the influences for choice of the railway as a suicide method. Together with this focus, it is essential to consider a range of geographical, physical, social and economic factors such as number of alcohol outlets and alcohol consumption level in a geographical area. Policy makers and railway organization owners should be encouraged to implement preventive measures that are evidence-based and tailor to their local structure of railway networks.

## Competing interests

The authors declare that they have no competing interests.

## Authors’ contributions

LST, AM, and RM contributed to the conception and design of the review. LST performed the literature searches, identified all potential articles and screened abstracts. LST and AM assessed full-text articles for eligibility and inclusion. LST and RM reviewed full-text of the papers that met the inclusion criteria and extracted the relevant information. LST prepared the early draft of the paper, and all authors commented on and refined the draft. All authors read and approved the final manuscript.

## Pre-publication history

The pre-publication history for this paper can be accessed here:

http://www.biomedcentral.com/1471-2458/14/20/prepub

## Supplementary Material

Additional file 1 **PRISMA checklist.** It has 27 items relevant to the content of a systematic review, which includes the title, abstract, methods, results, discussion and funding. It reports the page number where each item can be found in the review. Click here for file
